# The Over-Expression of Two Transcription Factors, ABS5/bHLH30 and ABS7/MYB101, Leads to Upwardly Curly Leaves

**DOI:** 10.1371/journal.pone.0107637

**Published:** 2014-09-30

**Authors:** Rui An, Xiayan Liu, Rui Wang, Haicui Wu, Shuang Liang, Jingxia Shao, Yafei Qi, Lijun An, Fei Yu

**Affiliations:** State Key Laboratory of Crop Stress Biology in Arid Areas and College of Life Sciences, Northwest A&F University, Yangling, Shaanxi, People's Republic of China; Universidad Miguel Hernández de Elche, Spain

## Abstract

Proper leaf development is essential for plant growth and development, and leaf morphogenesis is under the control of intricate networks of genetic and environmental cues. We are interested in dissecting these regulatory circuits genetically and report here the isolation of two Arabidopsis dominant mutants, *abnormal shoot5-1D* (*abs5-1D*) and *abs7-1D* identified through activation tagging screens. Both *abs5-1D* and *abs7-1D* display an intriguing upwardly curly leaf phenotype. Molecular cloning showed that the elevated expression of a bHLH transcription factor ABS5/T5L1/bHLH30 or a MYB transcription factor ABS7/MYB101 is the cause for the abnormal leaf phenotypes found in *abs5-1D* or *abs7-1D*, respectively. Protoplast transient expression assays confirmed that both ABS5/T5L1 and ABS7/MYB101 are targeted to the nucleus. Interestingly, the expression domains of auxin response reporter *DR5::GUS* were abnormal in leaves of *abs5-1D* and *ABS5*/*T5L1* over-expression lines. Moreover, cotyledon venation analysis showed that more areoles and free-ending veins are formed in *abs5-1D*. We found that the epidermis-specific expressions of *ABS5*/*T5L1* or *ABS7*/*MYB101* driven by the Arabidopsis *Meristem Layer 1* promoter (*P_AtML1_*) were sufficient to recapitulate the curly leaf phenotype of *abs5-1D* or *abs7-1D*. In addition, *P_AtML1_::ABS5* lines exhibited similar changes in *DR5::GUS* expression patterns as those found in 35S-driven *ABS5*/*T5L1* over-expression lines. Our work demonstrated that enhanced expressions of two transcription factors, ABS5/T5L1 and ABS7/MYB101, are able to alter leaf lamina development and reinforce the notion that leaf epidermis plays critical roles in regulating plant organ morphogenesis.

## Introduction

A major difference between plant and animal development is the *de novo* formation of plant organs such as leaves in post-embryonic development [1]. Advances in the past decade have uncovered elaborate regulatory pathways governing the morphing of pluripotent cells in plant apical meristems, both the shoot apical meristem (SAM) and the root apical meristem, into distinct organs [2]–[4]. For example, the proper establishment of a leaf is under the control of intricate networks of genetic pathways and environmental cues [5], [6]. As leaf primordia are emerging from the SAM, these pathways and factors work in concert to ensure the coordinated development of leaf primordia along three dimensions: the proximo-distal, the medio-lateral, and the adaxial-abaxial axes, into leaves that show asymmetric features along these axes [6].

In most plants, one key aspect of leaf development is the proper coordination of adaxial and abaxial growth to maintain relatively flat leaves that are maximized for photosynthesis [6], [7]. A growing list of genetic factors regulates the establishment of leaf adaxial and abaxial identities [6], [7]. The class III homeodomain-leucine zipper (HD-ZIP) transcription factors genes *PHABULOSA* (*PHB*), *PHAVOLUTA* (*PHV*), and *REVOLUTA* (*REV*) are factors that promote the adaxial fate [7]–[10]. These genes were first identified through gain-of-function mutants in which leaves show adaxialization, and loss-of-function mutations of *PHB, PHV* and *REV* show reduced adaxial fate and concurrently an abaxialization of leaves [7]–[10]. On the other hand, at the abaxial side of the leaf, a group of factors antagonistic with the HD-ZIPs work to determine the abaxial fate [7], [11]. These include *KANADI* (*KAN*) family transcription factors and epigenetic regulation through microRNA165/166 [12], [13]. In addition, the *YABBY* (*YAB*) genes, *ASYMMETRIC LEAVES1* (*AS1*) and *AS2* genes have also been demonstrated to participate in leaf adaxial-abaxial polarity determination [14]–[16].

Curly leaf mutants are one group of mutants that show aberrant abaxial-adaxial growth coordination, giving rise to upward or downward leaf curvature. The classical *Antirrhinum* mutant *cincinnata* clearly demonstrated that leaf surface curvature is under genetic regulation [17]. Indeed, genetic works in Arabidopsis have identified a number of curly leaf mutants, such as the *incurvata* (*icu*) series of mutants [18], [19]. Many genes defined by these mutants are potential regulators of gene expression at epigenetic, transcriptional or post-transcriptional levels [20]. The *icu1* mutant, also isolated as *curly leaf*, is defective in a polycomb-group gene involved in chromatin remodeling while the *ICU2* encodes the putative catalytic subunit of the eukaryotic type DNA polymerase α [20], [21]. Consistent with a role of microRNA in regulating leaf development, microRNA related mutations can also lead to leaf curling phenotypes. For instance, *HASTY*/*ICU3* codes for a member of the importin-β family nucleocytoplasmic transport receptors that might be involved in the nuclear export of microRNAs [22]. Genetically dominant curly leaf mutant have also been reported. Gain-of-function mutations in Class III HD-ZIP transcription factor gene *AtHB15*, alternatively known as *ICU4* or *CORONA*, caused upwardly curly leaves due to mutations in its microRNA processing site [23]. Phytohormone auxin also participates in the regulation of abaxial-adaxial polarity. The over-expression of Arabidopsis *IAMT1*, which encodes an indole-3-acetic acid (IAA) carboxyl methyltransferase that presumably converts active auxin IAA to inactive methyl-IAA ester, caused dramatic hyponastic leaf phenotypes [24]. Moreover,mutations that are impaired in the auxin induced degradation of AUX/IAA proteins could also lead to curly leaves [25]–[27]. The fact that numerous factors have been shown to be able to modulate leaf curvature suggests that higher plants utilize complex regulatory schemes to ensure the proper development of leaves.

Taking a genetic approach, we have identified and characterized factors that are involved in the regulation of plant leaf and shoot development [28]–[29]. Here, we report the identification of two upwardly curly leaf mutants in Arabidopsis, designated *abs5-1D* (*abnormal shoot5-1Dominant*) and *abs7-1D*. We cloned *ABS5* and *ABS7* and demonstrated that *ABS5* encodes a bHLH transcription factor bHLH30 and *ABS7* encodes a MYB transcription factor MYB101, and both ABS5 and ABS7 were targeted into the nucleus. Interestingly, auxin homeostasis and leaf venation development were altered in *abs5-1D*. We assayed potential transcriptional activation activities of ABS5 and ABS7 and found that ABS7 is capable of activating reporter gene expression, while ABS5 alone is not. We further showed that the expression of *ABS5* or *ABS7* specifically in the epidermis was sufficient to cause leaf curvature similar to those of *abs5-1D* and *abs7-1D*, reconfirming the importance of epidermis in regulating leaf development. Although the phenotypes of *abs5-1D* and *abs7-1D* were results of ectopically expressed genes, our work do demonstrate the utilities of gain-of-function genetic approaches in uncovering potential regulators of plant development and these two genes may be exploited in the future for generating curly leaf traits when desired.

## Results

### The isolation of a dominant curly leaf mutant, *abs5-1D*

**Figure 1:**
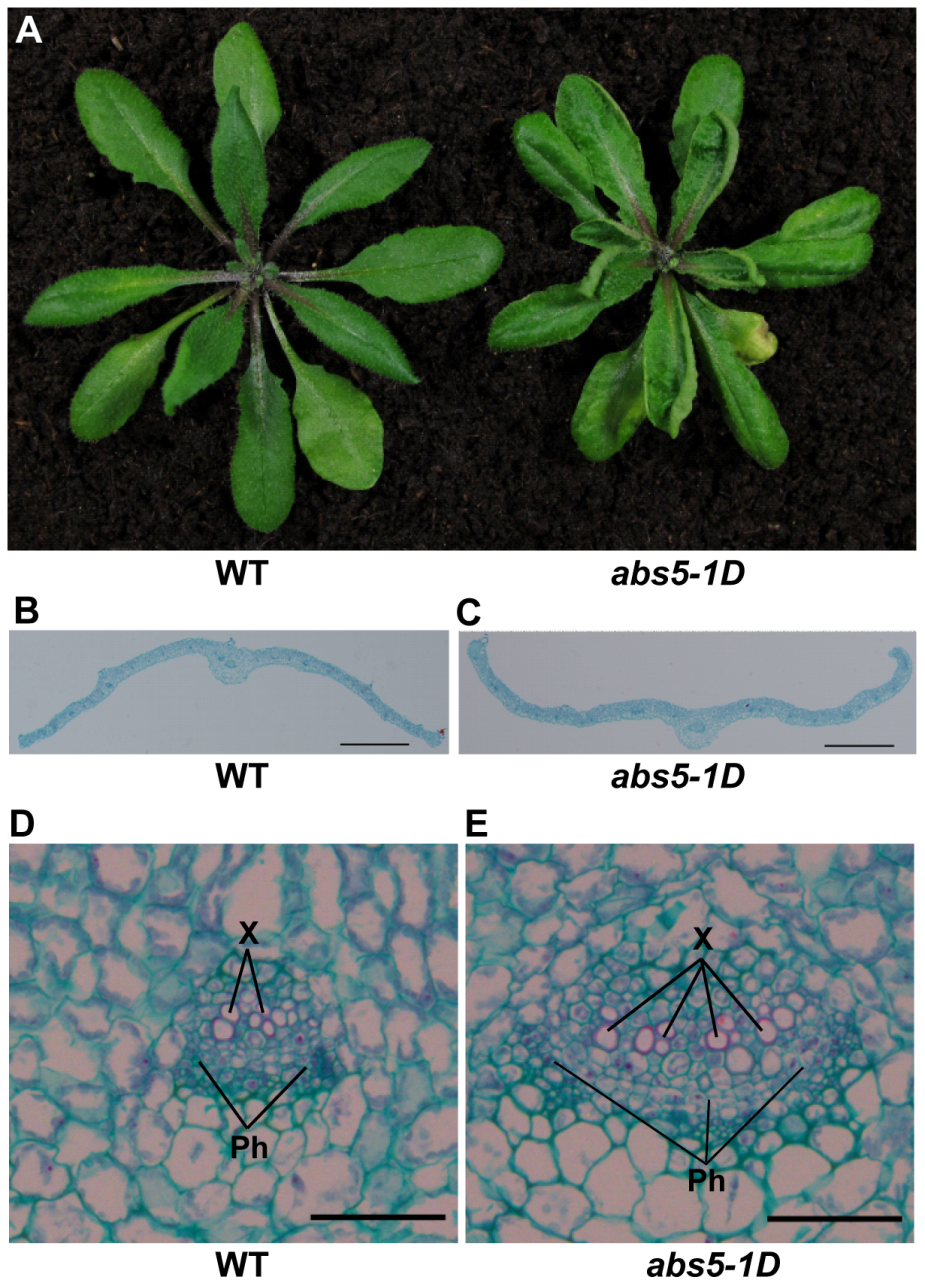
Phenotypes of *abs5-1D*. **A**. Leaf rosettes of five-week-old wild type and *abs5-1D* mutant. To have a clear view of the rosette leaves, the inflorescence stems were removed prior to photographing. **B–C**. Overview of the transverse sections of the eighth rosette leaf from three-week-old wild type (**B**) and *abs5-1D* (**C**). Bars: 500 µm. **D–E**. Transverse sections of the mid-vein regions of wild type (**D**) and *abs5-1D* (**E**) leaf. Bars: 50 µm.

We are interested in the regulatory schemes that ensure the proper development of plant leaves and have carried out genetic screens for mutants with altered leaf and shoot morphologies [28]–[30]. Through screening activation-tagged Arabidopsis mutant pools, we identified a dominant leaf development mutant and designated it *abnormal shoot5-1D* (*abs5-1D*; D for dominant) (Figure 1A). The most prominent phenotype of *abs5-1D* is the upward curling of leaf margins, in contrast to the slightly downward curvature usually observed in wild type (Figure 1A–B). Examination of the transverse sections of the eighth rosette leaves from five-week-old wild type and *abs5-1D* plants confirmed our visual observations (Figure 1B–C). Closer examination of leaf anatomy revealed that although the general arrangements of the palisade and spongy mesophyll cells are not grossly changed in *abs5-1D*, the number of cells composing the vascular bundles were increased in *abs5-1D* compared with that of wild type (Figure 1D–E). Moreover, there were also defects associated with floral development in *abs5-1D*, namely an increase in the number of secondary inflorescences (Table 1). Taken together, these observations suggest that the mutation in *abs5-1D* leads to pleiotropic developmental defects.

**Table 1:** Comparison of the average number of secondary inflorescences of wild type, *abs5-1D* and *ABS5*/*T5L1* OE lines.

Genotype	Average Number of Secondary Inflorescences	Number of Plants Scored
WT	3.44±0.11	25
*abs5-1*D	4.60±0.14**	25
*ABS5* OE-5	4.64±0.15**	25
*ABS5* OE-10	5.00±0.17**	25

Data were presented in the form of mean±standard error (SE). Differences between wild type and each of the mutant lines were evaluated by a *p*-value generated by one-sided *t*-test (**: *p*<0.01).

### The up-regulation of At1g68810 causes *abs5-1D* phenotypes

**Figure 2:**
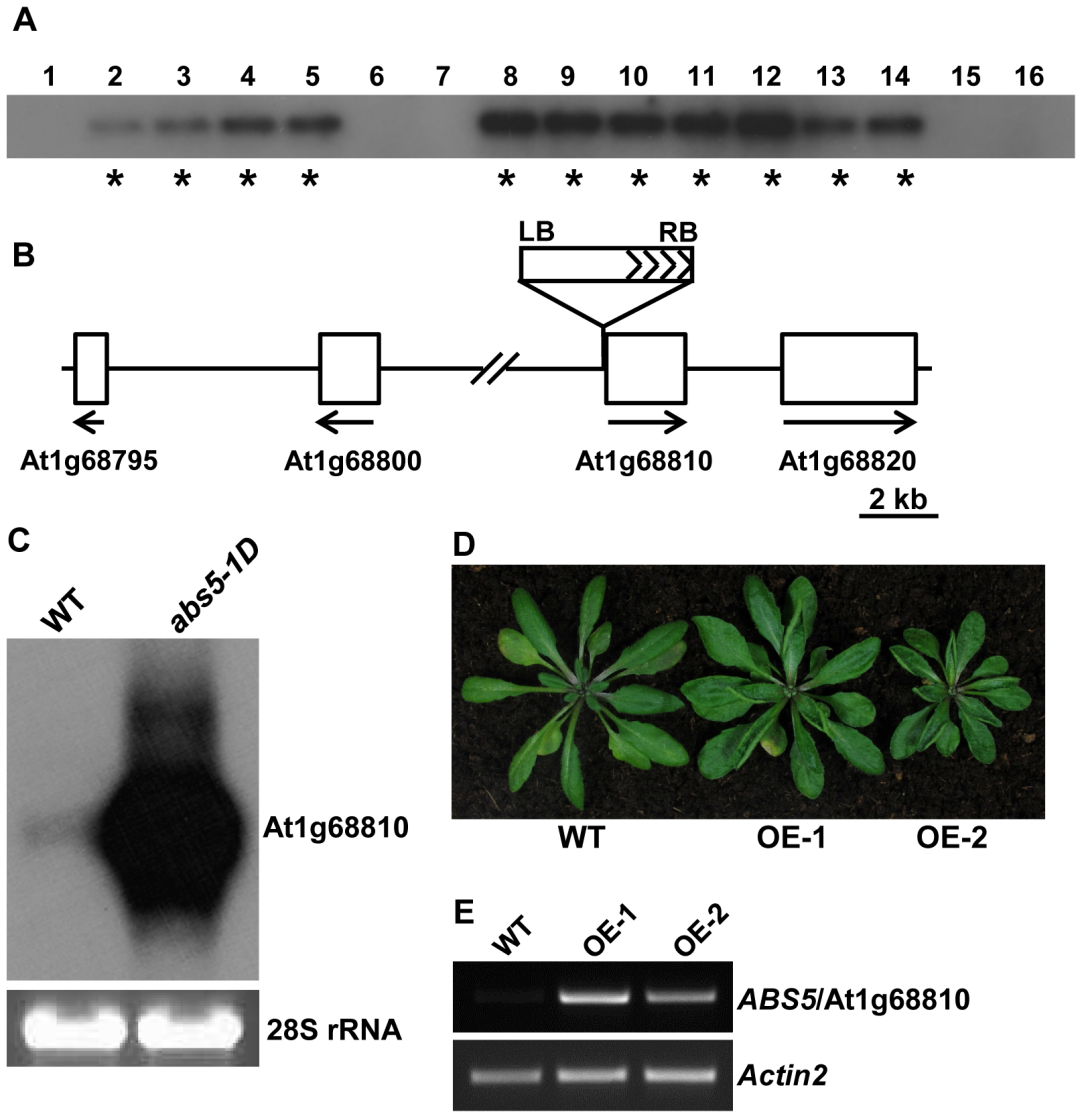
Cloning of *ABS5* gene. **A**. Co-segregation analysis of *abs5-1D*. Genomic DNAs were extracted from 16 F2 progenies from a cross between wild type and *abs5-1D*. DNA samples were digested with *Hind*III and separated on an agarose gel before transferred onto a nylon membrane. The DNA gel blot was probed with ^32^P-labelled *BAR* gene sequences. Plants with *abs5-1D*-like phenotypes were marked with asterisks. **B**. Schematic representation of the T-DNA insertion site in *abs5-1D*. Open boxes represent genes in the vicinity of the activation tagging T-DNA. Solid lines represent intergenic regions. The orientations of these genes' open reading frames (ORFs) were indicated by arrows. **C**. Accumulation of At1g68810 transcripts in wild-type and *abs5-1D*. Equal amounts of total RNA (5 µg) extracted from 2-week-old seedlings were separated on a formaldehyde gel and transferred to a nylon membrane. The blot was hybridized with ^32^P-labelled full-length At1g68810 cDNA. The ethidium bromide-stained RNA gel served as a loading control. **D**. Leaf rosettes of representative five-week-old wild type and two independent At1g68810 over-expression lines (OE-1 and OE-2). **E**. Semi-quantitative RT-PCR analysis of At1g68810 transcripts accumulation in the plants shown in (**C**).

Since *abs5-1D* was isolated from activation-tagged T-DNA mutant pools, we tested whether *abs5-1D* phenotypes co-segregated with T-DNA insertion(s). Southern blot analysis of 16 F2 progenies from a cross between *abs5-1D* and wild type showed that a single T-DNA insertion was detected in all the plants showing an *abs5-1D*-like phenotype, indicating a close linkage between the *abs5-1D* mutation and the T-DNA insertion (Figure 2A). We next recovered the plant genomic sequences flanking the T-DNA insertion site via plasmid rescue. Blast search against the Arabidopsis whole genome sequences revealed that the activation T-DNA was inserted in the intergenic region between genes At1g68800 and At1g68810 (Figure 2B). The T-DNA right border was 204 bp upstream of the At1g68810 start codon. In addition, northern blot analysis showed that the accumulation of At1g68810 transcripts was greatly increased in *abs5-1D* compared to that of wild type (Figure 2C). To confirm that the over-expression of At1g68810 led to the *abs5-1D* phenotypes, a vector harboring a full-length cDNA of At1g68810 under the control of the constitutive cauliflower mosaic virus 35S promoter was constructed and transformed into wild type Arabidopsis. Independent transgenic lines recapitulated the leaf curling up phenotypes of *abs5-1D* in T1 and T2 generations (Figure 2D). The up-regulation of At1g68810 in these over-expression (OE) lines was confirmed by semi-quantitative RT-PCR (Figure 2E). Moreover, At1g68810 OE lines also showed increased secondary inflorescence numbers (Table 1). Taken together, these data established that the developmental phenotypes associated with *abs5-1D* mutation are due the enhanced expression of At1g68810 and *ABS5* gene is At1g68810. At1g68810 was previously identified as *TMO5-LIKE1* (*T5L1*) and was implicated in the regulation of vascular tissue development [31].

### *ABS5*/*T5L1* encodes a putative bHLH transcription factor

*ABS5* is annotated to encode a protein of 368 amino acids and protein sequence analysis revealed that ABS5 is likely a putative transcription factor belonging to the basic helix-loop-helix (bHLH) family [32]. In Arabidopsis, there are at least 147 members in the bHLH family and ABS5/T5L1 was previous annotated as bHLH30 [32].

**Figure 3:**
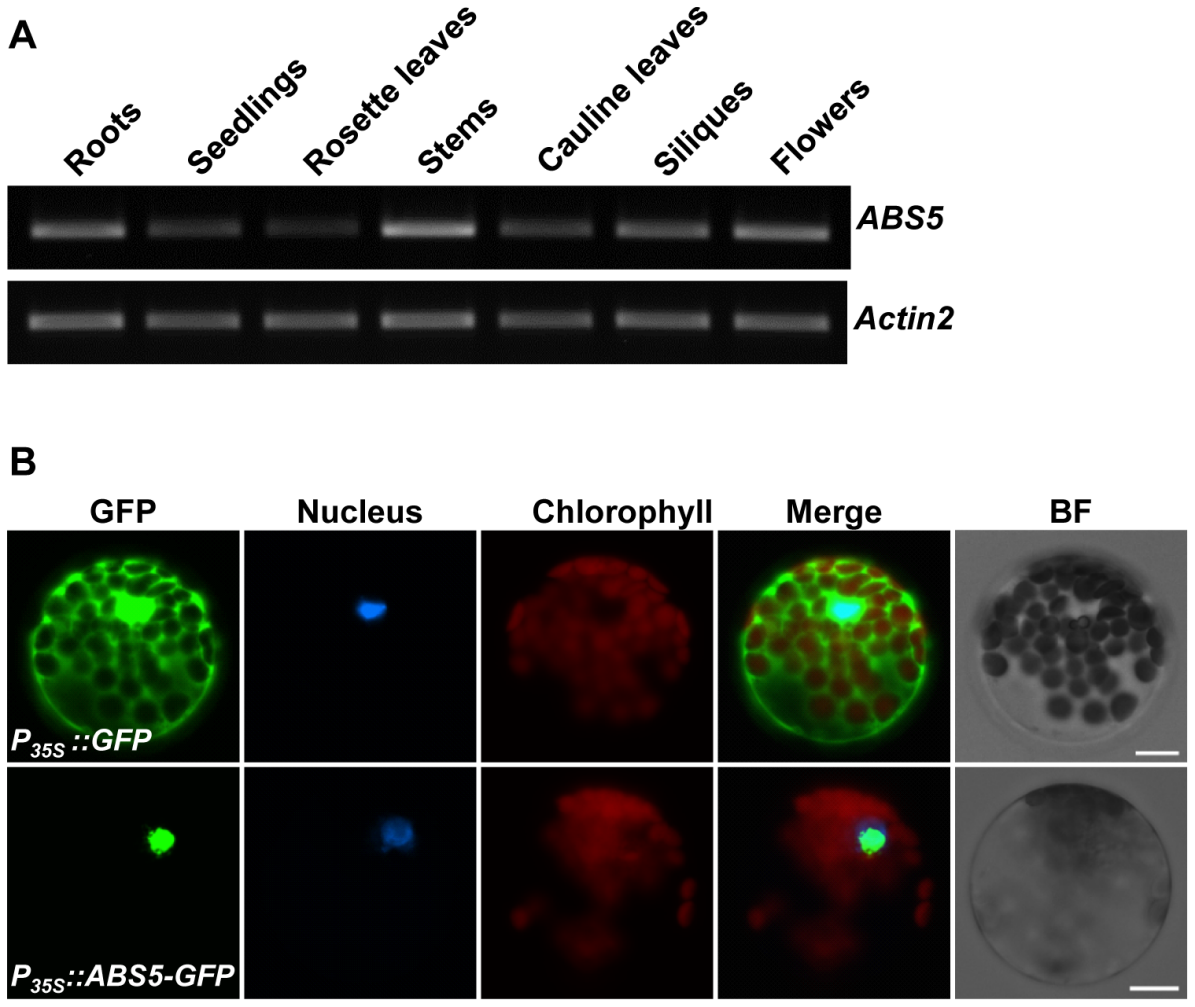
Expression analysis of *ABS5*/*T5L1*. **A**. Semi-quantitative RT-PCR analysis of *ABS5*/*T5L1* gene expression in various tissues: roots, two-week-old seedlings, rosette leaves, stems, cauline leaves, siliques and flowers. The expression of *Actin2* was used as a control. **B**. Nuclear localization of ABS5-GFP fusion protein in Arabidopsis leaf protoplasts. Wild type leaf protoplasts were transformed with *P_35S_::GFP* or *P_35S_::ABS5-GFP*. A single representative protoplast was shown for each transformation. Nuclei were specifically stained by the fluorescent dye Hoechst33342. Hoechst33342, GFP, chlorophyll autofluorescence signals were monitored by fluorescence microscopy. Bright field (BF) images served as controls for protoplast integrity. Bars: 10 µm.

As an initial attempt to understand the function of *ABS5*/*T5L1*, we examined its tissue expression profile via semi-quantitative RT-PCR with cDNAs obtained from various wild type Arabidopsis tissues. Figure 3A shows that *ABS5* transcripts accumulated in all tissues examined. *ABS5*/*T5L1* expression was relatively lower in aerial part of two-week-old seedlings and older rosette leaves but is highly expressed in roots and stems (Figure 3A). We next explored the sub-cellular localization of ABS5/T5L1 protein. Vectors expressing eGFP alone or the ABS5-GFP fusion protein under the control of 35S promoter were used to transform wild type leaf protoplasts. Nuclei of protoplasts were labeled via staining with the fluorescent dye Hoechst33342 [33]. Protoplasts expressing the control *P_35S_::GFP* showed GFP signals in both the cytosol and the nucleus (Figure 3B). In contrast, protoplasts expressing *P_35S_::ABS5-GFP* displayed green fluorescence signals exclusively in the nucleus, as indicated by the Hoechst33342 staining (Figure 3B). These data show that ABS5/T5L1 resides in the nucleus, consistent with its potential function as a transcription factor.

### *ABS5*/*T5L1* over-expression alters auxin homeostasis and cotyledon vein patterns

**Figure 4:**
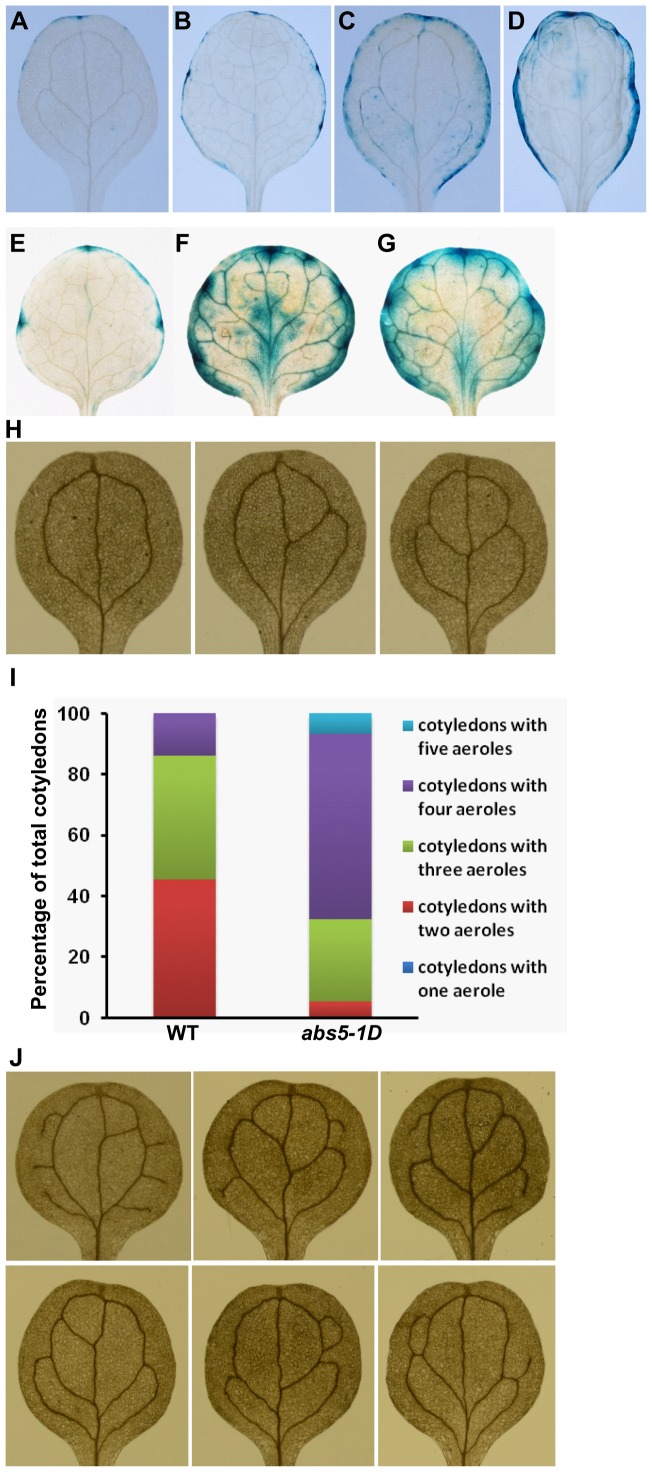
Comparisons of *DR5::GUS* activities and cotyledon vein patterns between wild type and *abs5-1D*. **A–D**. *DR5::GUS* activities in heterozygous *DR5::GUS* (**A** and **B**) and *DR5::GUS abs5-1D*/+ double heterozygous backgrounds (**C** and **D**). Illustrated are representative cotyledons (**A** and **C**) and the first true leaves (**B** and **D**) from two-week-old seedlings. **E–G**. *DR5::GUS* activities in the first true leaves of two-week-old homozygous *DR5::GUS* lines (**E**) and two independent 35S promoter driven *ABS5*/*T5L1* OE lines in *DR5::GUS* background (**F** and **G**). **H**. Representative cotyledon vein patterns in wild type. Cotyledons with two, three, or four areoles were observed in wild type [35]. **I**. Quantification of cotyledon vein patterns in 10-day-old wild type (n = 423) and *abs5-1D* (n = 412). **J**. Abnormal cotyledon vein patterns observed in *abs5-1D*. Illustrated are cotyledons with abnormal tertiary veins (top row) and cotyledons with five areoles (bottom row).

Given that auxin plays a key role in leaf morphogenesis we next examined whether auxin homeostasis is altered in *abs5-1D*. The expression patterns of the synthetic *DR5::GUS* reporter gene were used to deduce the distributions of auxin maxima [34]. In wild type background, the strongest *DR5::GUS* signals coincided with the positions of the hydathodes in cotyledons and the first true leaves of two-week-old seedlings (Figure 4A–B). However, in *abs5-1D*/+ heterozygous background, *DR5::GUS* activities were less restricted but more evenly distributed along the entire leaf margin compared to that of wild type (Figure 4C–D). As illustrated in Figure 4E–G, stronger and more diffused GUS signals were also found in leaf marginal areas in transgenic lines over-expressing *ABS5*/*T5L1* in *DR5::GUS* background. These observations led us to investigate whether other auxin related processes are also disturbed in *abs5-1D*. Previous studies have implicated that both the initiation and differentiation of vascular strands are regulated by auxin transport and signaling in leaves [35], [36]. Since Arabidopsis cotyledons display simple and predictable patterns of vasculature development, we compared mature cotyledon vein patterns of wild type and *abs5-1D*. Under our growth conditions, wild type cotyledons predominantly displayed two, three or four areoles (45.1%, 40.9% and 13.8%, respectively) (Table 2; Figure 4H) [35]. In contrast, *abs5-1D* cotyledons displayed a different distribution of cotyledon vein patterns (Table 2; Figure 4I–J). The proportions of cotyledons with four or more areoles were increased while those with two or three areoles were decreased in *abs5-1D* (Table 2; Figure 4I). Notably, 6.8% of *abs5-1D* cotyledons developed veins with five areoles, which is usually not seen in wild type (Figure 4I–J). In addition, in *abs5-1D* cotyledons with three or four areoles, vein patterns were usually more complex than those of wild type, due to the presence of multiple free-ending tertiary veins (Figure 4J). Taken together, these data suggested that auxin homeostasis and vascular development are likely perturbed by the *abs5-1D* mutation.

**Table 2:** Quantification of cotyledon vein patterns in wild type and *abs5-1D*.

		Cotyledon Vein Patterns				
Genotype	Total*	One Areole	Two Areoles	Three Areoles	Four Areoles	Five Areoles
WT	423	1 (0.2%)	191 (45.1%)	173 (40.9%)	58 (13.8%)	N.A.
*abs5-1D*	412	N.A.	22 (5.3%)	112 (27.2%)	250 (60.7%)	28 (6.8%)

Ten-day-old wild type or *abs5-1D* seedlings were de-colored with 70% ethanol and examined under a Nikon SMZ1500 stereoscope. Cotyledon vein patterns were scored based on the number of areoles formed.

*total numbers of cotyledons examined for genotype.

### The identification of a second dominant curly leaf mutant, *abs7-1D*

**Figure 5:**
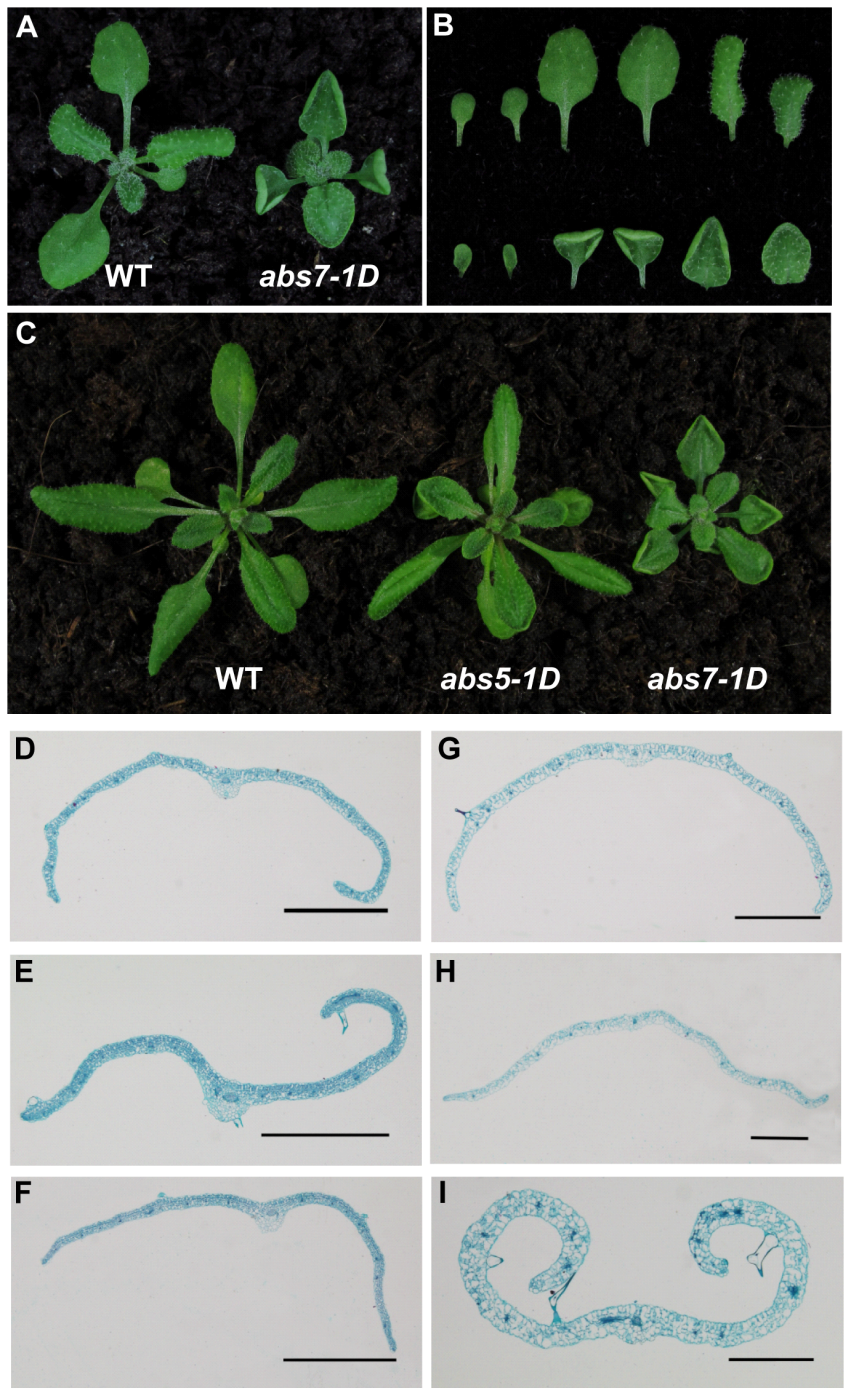
Isolation of *abs7-1D*. **A**. Phenotypes of representative two-week-old wild type and *abs7-1D* seedlings. **B**. Comparison of individual leaves detached from plants shown in (**A**). From left to right are the two cotyledons and the first four rosette leaves (Upper panel: wild type; Lower panel: *abs7-1D*). **C**. Comparison of the overall plant statues of three-week-old wild type, *abs5-1D* and *abs7-1D*. **D-F**. Transverse sections of the ninth rosette leaves of three-week-old wild type (**D**), *abs5-1D* (**E**) and *abs7-1D* (**F**). **G–I**. Transverse sections of the first rosette leaves of three-week-old wild type (**G**), *abs5-1D* (**H**) and *abs7-1D* (**I**).

During the course of our work, we isolated another curly leaf mutant, which was designated *abs7-1D*, also from our activation tagging T-DNA mutant pools (Figure 5A). Overall *abs7-1D* displayed upwardly curly leaf phenotypes that were reminiscent of *abs5-1D* (Figure 5A–B). However, there are several distinctions between the two mutants. First, the overall plant stature of homozygous *abs7-1D* was considerably smaller than that of wild type while the size of *abs5-1D* is comparable to that of wild type (Figure 5C). Second, the timing of leaf curling is different in *abs5-1D* and *abs7-1D* (Figure 5C–I). In *abs5-1D*, the upward leaf curling was more obvious in newly emerged young leaves while old leaves were only slightly curled up in marginal areas (Figure 5E,H). On the contrary, in *abs7-1D* young leaves at the center of the rosette were not curled up, the upwardly curling leaf phenotype was more conspicuous in mature leaves in *abs7-1D* (Figure 5C,F,I). To understand the cellular basis of the “curly leaf” phenotypes in *abs5-1D* and *abs7-1D*, we measured the average number and length of abaxial and adaxial epidermal cells in wild type and mutants at the developmental stages when their “curly leaf” phenotypes were most obvious (Figure S1). Statistical analysis of the measurements showed that the number of epidermal cells on either the adaxial or the abaxial side of the leaves was about the same in *abs5-1D* or *abs7-1D* compared to that of wild type, suggesting epidermal cell proliferation was not grossly altered in *abs5-1D* or *abs7-1D* (Figure S1A, C). On the other hand, although the average length of abaxial epidermal cells of *abs5-1D* or *abs7-1D* was comparable to that of wild type, the average length of adaxial epidermal cells of *abs5-1D* or *abs7-1D* was significantly reduced compared to that of wild type (Figure S1B, D). These observations suggest that the “upwardly curly leaf” phenotypes in *abs5-1D* or *abs7-1D* is probably due to more restricted expansion of leaf epidermal cells on the adaxial side.

**Figure 6:**
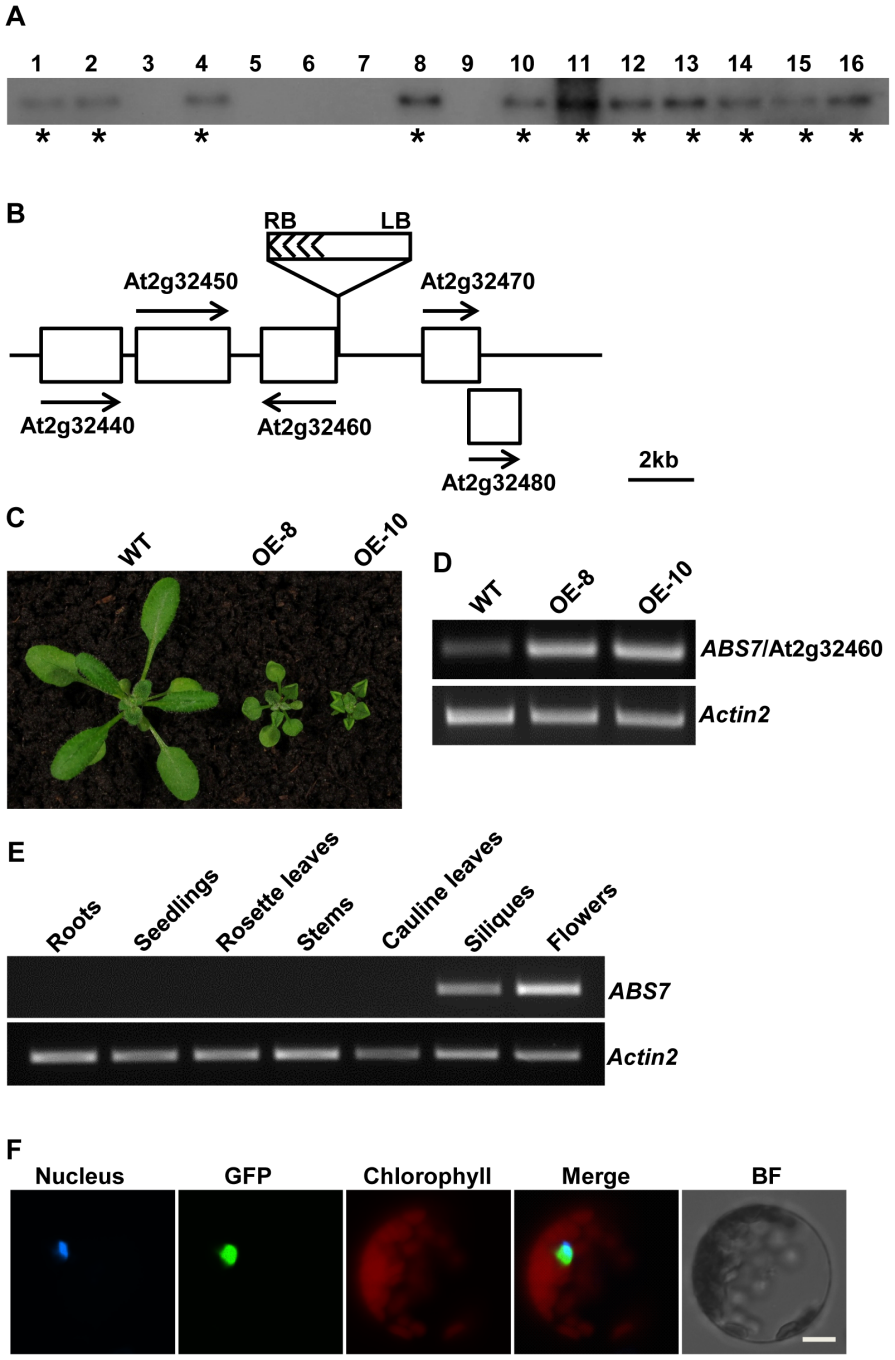
Cloning of *ABS7* gene. **A**. Co-segregation analysis of *abs7-1D*. 16 randomly selected plants from the F2 progeny from a cross between wild type and *abs7-1D* were used for analysis. Southern blot analysis was performed as in Figure 2A. Plants with *abs7-1D*-like phenotypes were marked by asterisks. **B**. Schematic representation of the T-DNA insertion site in *abs7-1D*. Genes in the vicinity of the T-DNA insertion site were represented by white boxes. Arrows indicated the orientation of the ORFs of these genes. **C**. Phenotypes of representative three-week-old wild type and two *ABS7* over-expression lines (OE-8 and OE-10). **D**. Expression levels of *ABS7* gene in wild type and two *ABS7* OE lines analyzed via semi-quantitative RT-PCR. **E**. Semi-quantitative RT-PCR analysis of *ABS7* gene expression in roots, two-week-old seedlings, rosette leaves, stems, cauline leaves, siliques and flowers. The expression of *Actin2* was used as a control. **F**. Nuclear localization of ABS7-GFP fusion protein in Arabidopsis leaf protoplasts. Protoplast transformation, nucleus staining and fluorescence microscopy was performed as in Figure 3B.

The leaf phenotypes of *abs7-1D* co-segregated with T-DNA insertion(s) (Figure 6A). Through plasmid rescue, we identified a T-DNA insertion 102 bp upstream of the start codon of At2g32460 (Figure 6B). Given the dominant nature of *abs7-1D*, we tested whether the over-expression of At2g32460 was the cause for curly leaves in *abs7-1D*. Figure 6C shows that independent At2g32460 OE lines phenocopied *abs7-1D* and the up-regulations of At2g32460 in these lines were confirmed via semi-quantitative RT-PCR (Figure 6D). These results indicate that enhanced expression of At2g32460 underlines the leaf curling up phenotypes of *abs7-1D* and *ABS7* is At2g32460. *ABS7* encodes a member of the Arabidopsis MYB family transcription factors and was designated MYB101 [37]. Phylogenetic studies have shown that ABS7/MYB101 and four other MYB transcription factors (MYB33, MYB65, MYB97 and MYB120) belong to a small family called the GAMYBs [37]. We next analyzed the accumulation of *ABS7*/*MYB101* transcripts in different Arabidopsis tissues. As shown in Figure 6E, *ABS7*/*MYB101* transcripts were only detected in flowers and siliques by semi-quantitative RT-PCR. This is consistent with previous finding that *ABS7*/*MYB101* is highly expressed in seeds and floral tissues [38]. Consistent with its identity as a transcription factor, ABS7-GFP localized to the nucleus in protoplast transient expression assays (Figure 6F). These results suggested that although *ABS7*/*MYB101* is not normally expressed in leaves, it is able to change leaf morphology when artificially over-expressed.

### The isolation of loss-of-function mutations in *ABS5*/*T5L1* and *ABS7*/*MYB101*

To further examine the roles that *ABS5*/*T5L1* and *ABS7*/*MYB101* play in plant development, we sought for loss-of-function alleles of *ABS5*/*T5L1* and *ABS7*/*MYB101*. A transposon tagged line (SM_3_20727) and a T-DNA insertional line (SALK_149918) were obtained from ABRC for *ABS5*/*T5L1* and *ABS7*/*MYB101*, respectively [39], [40]. PCR and sequencing analysis confirmed that the transposon was inserted in the 5′ untranslated region (UTR) of *ABS5*/*T5L1*, 19 bp upstream of its start codon, and the homozygous line was named *abs5-1* (Figure S2A–B). Semi-quantitative RT-PCR analysis showed that the accumulation of *ABS5*/*T5L1* transcripts was reduced in *abs5-1* (Figure S2C). Under our growth conditions, we did not observe major developmental abnormalities with *abs5-1*, suggesting that the partial loss of *ABS5*/*T5L1* is not detrimental to plant growth (Figure S2D).

For the putative *ABS7*/*MYB101* knockout line SALK_149918, T-DNA was confirmed to be inserted in the second exon of *ABS7*/*MYB101*, 1408 bp downstream of the start codon, and the homozygous line was named *abs7-1* (Figure S3A–B). Full-length *ABS7*/*MYB101* transcripts were not detected in *abs7-1* (Figure S3C). However, *abs7-1* plants were indistinguishable from wild type plants, suggesting *ABS7*/*MYB101* is dispensable for normal plant growth and development, at least under lab conditions and there might be additional genes sharing redundant functions with *ABS7*/*MYB101* (Figure S3D).

Since the *ICU* genes are known regulators of leaf curvature [19]–[23], we next tested whether the “upwardly curly leaf” phenotype in *abs5-1D* and *abs7-1D* is related to changes of the expression levels of *ICU* genes. We compared the accumulation of *ICU1*, *ICU2*, *ICU3* and *ICU4* transcripts in wild type, loss-of-function and activation-tagged lines of *ABS5*/*T5L1* and *ABS7*/*MYB101* using semi-quantitative RT-PCR. No significant changes in the expression levels of any *ICU* genes were observed in these plants (Figure S4A–B). These data suggested that the over-expression of *ABS5*/*T5L1* or *ABS7*/*MYB101* may influence leaf lamina developments via pathways that are not mediated by *ICU1-4* genes.

### Trans-activation activity assays of ABS5/T5L1 and ABS7/MYB101

**Figure 7:**
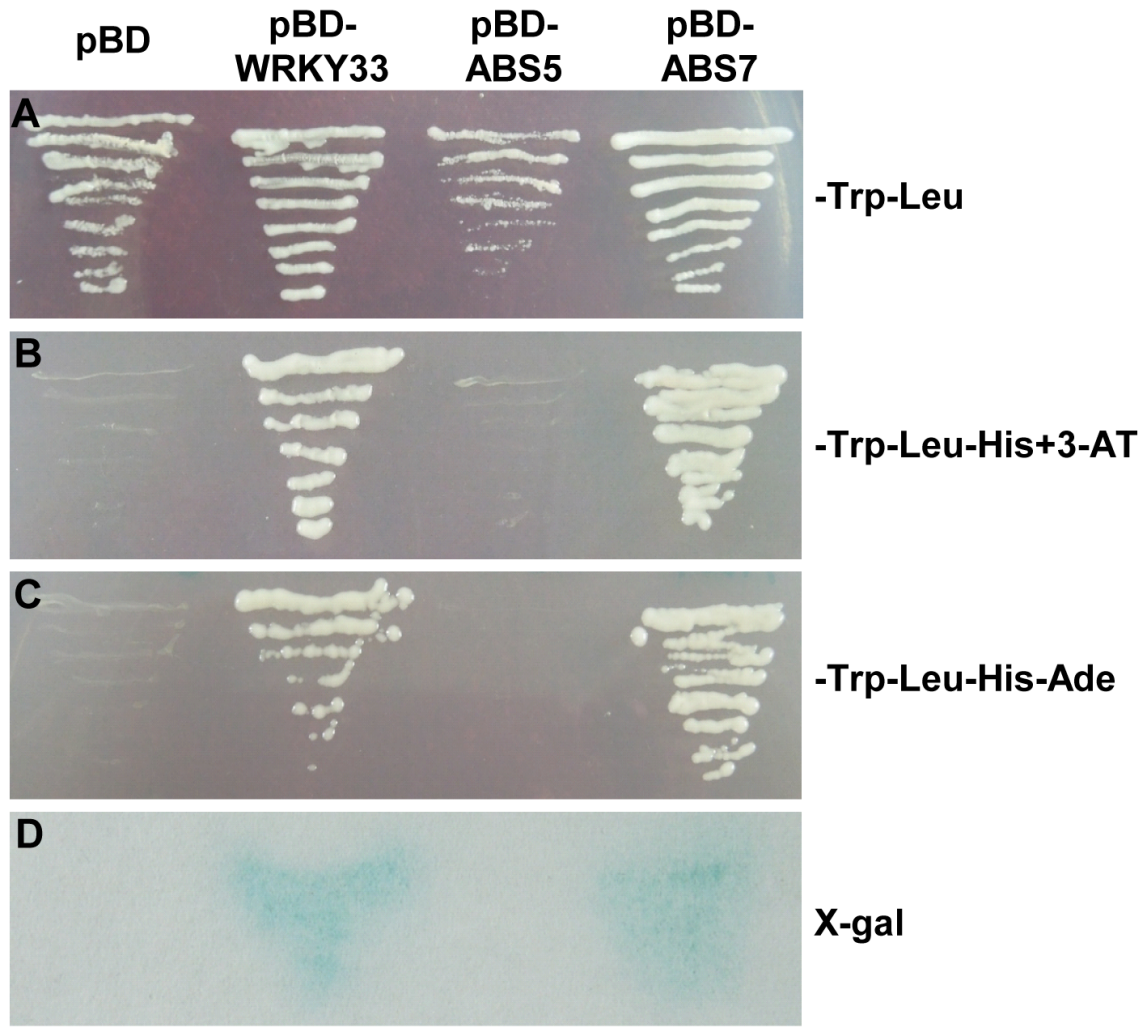
Transcriptional activation analysis of ABS5/T5L1 and ABS7/MYB101 in yeast. Yeast strain AH109 was transformed with a negative control vector (pBD), a positive control pBD-WRKY33, pBD-ABS5 or pBD-ABS7, respectively. Each of the BD vectors was co-transformed with an empty AD vector, pGADT7. **A–C**. Growth of yeast transformants on the SD/-Trp-Leu medium (**A**), the SD/-Trp-Leu-His medium plus 5 mM 3-AT (**B**) or the SD/-Trp-Leu-His-Ade medium (**C**). **D**. Activation of the *LacZ* gene analyzed via filter lifting X-β-gal assays.

To test whether ABS5/T5L1 and ABS7/MYB101 could function as transcription activators, we carried out trans-activation activity assays in yeast. The open reading frames of *ABS5* and *ABS7* were fused to the 3′ end of the GAL4 DNA binding domain (BD) to generate pBD-ABS5 and pBD-ABS7 vectors, respectively. The empty vector containing only the GAL4 DNA binding domain served as a negative control and Arabidopsis *WRKY33* gene was used as a positive control [41]. Each construct was co-transformed with pGADT7 into yeast strain AH109. The expression of three reporter genes, *HIS3*, *ADE* and *LacZ* were assayed. As expected, all yeast transformants grew on SD/-Trp-Leu medium (Figure 7A). However, only yeast transformants harboring pBD-ABS7 or the positive control vector were able to grow on SD/-Trp-Leu-His (w/5 mM 3-AT) or SD/-Trp-Leu-His-Ade media and gave positive results in X-gal assay, suggesting that ABS7/MYB101 is able to activate reporter genes (Figure 7B–D). On the other hand, yeast cells expressing pBD-ABS5 or the negative control vector failed to activate the reporter genes (Figure 7B–D). Taken together, these results indicate that ABS7/MYB101 protein has transcriptional activation activity, while ABS5/T5L1, at least when alone, may not possess transcriptional activation activities.

### Epidermal expression of *ABS5*/*T5L1* or *ABS7*/*MYB101* is sufficient to cause leaf curvature

**Figure 8:**
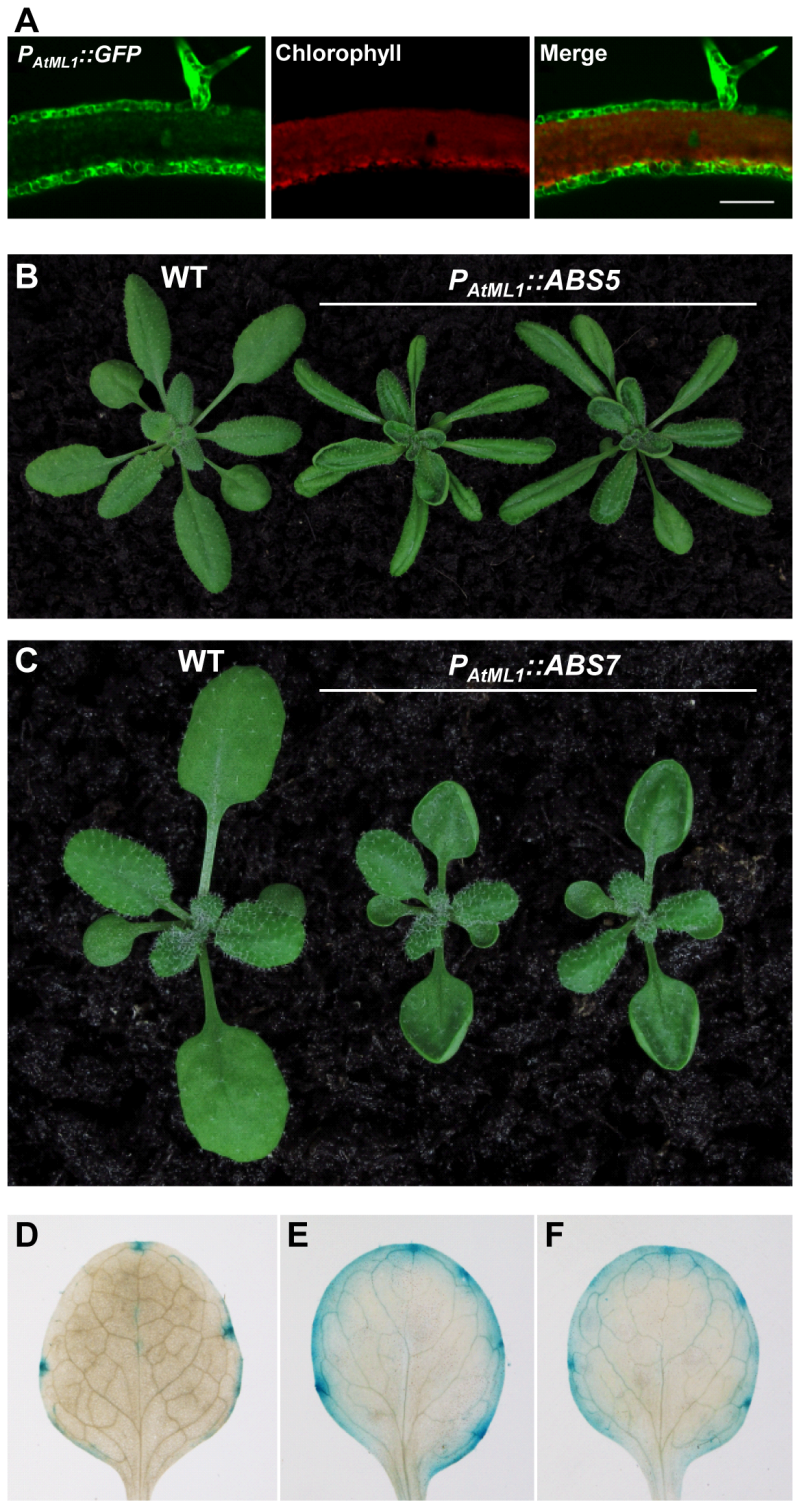
Effects of epidermal specific expression of *ABS5*/*T5L1* or *ABS7*/*MYB101*. **A**. Verification of the epidermal specific *AtML1* promoter (*P_AtML1_*). The transverse sections of young leaves from the *P_AtML1_::GFP* expressing Arabidopsis lines were examined via con-focal microscopy. Mesophyll cells were visualized through the chlorophyll autofluorescence. **B**. Phenotypes of three-week-old wild type and two independent *P_AtML1_::ABS5* lines. **C**. Phenotypes of two-week-old wild type and two independent *P_AtML1_::ABS7* lines. **D–F**. *DR5::GUS* activities in the first true leaves of two-week-old homozygous *DR5::GUS* line (**D**) and two independent lines expressing *P_AtML1_::ABS5* in *DR5::GUS* background (**E** and **F**).

Epidermis is an integral part of plant leaf and has been shown to regulate many aspects of plant growth and development [42]. To investigate the potential impact of *ABS5*/*T5L1* or *ABS7*/*MYB101* over-expression in the epidermis, fusion constructs with *ABS5*/*T5L1* or *ABS7*/*MYB101* cDNA under the control of epidermal layer specific Arabidopsis *Meristem Layer 1* promoter (*P_AtML1_*) were generated [43]. Transgenic lines harboring *P_AtML1_::GFP* were used to verify the epidermis-specific expression profile (Figure 8A). Interestingly, multiple lines that express *P_AtML1_::ABS5* showed the curly leaf phenotype, similar to that observed in *abs5-1D* (Figure 8B). Moreover, transgenic lines with epidermal-specific expressions of *ABS7*/*MYB101* phenocopied *abs7-1D* (Figure 8C). Next, we tested whether the epidermal- specific expression of *ABS5*/*T5L1* is sufficient to alter auxin homeostasis. We transformed *DR5::GUS* plants with *P_AtML1_::ABS5* construct and assayed GUS activities in independent transgenic lines that exhibited the upward curling leaf phenotype. Figure 8D-F showed that auxin distributions as indicated by the expressions of *DR5::GUS* were increased in *P_AtML1_::ABS5* lines in a way that is similar to what was found in *abs5-1D* or the *ABS5*/*T5L1* OE lines. Taken together, these results indicate that specific over-expression *ABS5*/*T5L1* or *ABS7*/*MYB101* in the epidermal layer alone was sufficient to alter leaf development, reinforcing the idea that the epidermis plays an important role in plant organ shape determination.

## Discussion

Leaf development is one of the fundamental processes ensuring robust photoautotrophic growth for higher plants and mechanisms are in place to coordinate the establishment of leaf polarities [6]. In this study, we report the isolation of two dominant leaf polarity mutants, *abs5-1D* and *abs7-1D*, both displayed an “upwardly curly leaf” phenotype (Figures 1 and 5).

We established that the over-expression of a bHLH transcription factor ABS5/T5L1 was responsible for the intriguing curly leaf phenotype in *abs5-1D*. Furthermore, we found that the homeostasis of phytohormone auxin, as indicated by the expression pattern of auxin reporter gene *DR5::GUS*, was also disturbed in *abs5-1D* mutants (Figure 4A-G). Auxin is a key regulator of leaf morphogenesis and vasculature development [44]. A number of Arabidopsis auxin signaling mutants display “curled up” leaf phenotype similar to that of *abs5-1D*. For example, *bodenlos* (*bdl*) mutant, the gain-of-function mutant allele of the *IAA12* gene, showed a leaf curling up phenotype [45]. Mutation in *bdl* allele dampens the auxin induced degradation of IAA12 protein via the ubiquitin-proteasome pathway [45]. Interestingly, mutations in several other IAA genes that have comparable impact on IAA proteins, including in *IAA3* (*shy2-2*), and *IAA17* (*axr3-3*, *axr3-1*and *icu6*) also give rise to similar defects in leaf morphology [46], [25], [26].

Consistent with disturbed auxin homeostasis and auxin's involvement in leaf vasculature development, we also determined that *abs5-1D* has abnormal cotyledon venation patterns (Figures 1 and 4). We found that both the complexity and the number of free ending veins were increased in *abs5-1D* cotyledons compared with those of wild type (Figure 4H–J). During leaf vasculature development, the canalization hypothesis indicates that the convergence of auxin polar transport to the tip of the developing leaf primordia and the subsequent inward flow of auxin is critical for the establishment of leaf vasculature [35], [36], [44]. The flow of auxin defines the expression domains of auxin efflux carrier PIN1, and the polarized PIN1 localization further enhances the polar transport of auxin [36]. In developing young leaves, both the differentiation of procambial cells and the formation of new vascular strands depend on auxin polar transport via PIN1 [36], [44]. On the other hand, genetic screens for mutants defective in vein patterns have also identified genes involved in auxin signaling [47], [48]. It is possible that altered auxin distribution in *abs5-1D* affects the polar auxin transport process, which in turn leads to abnormalities in vasculature development.

Previous studies have shown that *ABS5*/*T5L1* is the closest homolog of *TMO5*, a direct target of MONOPTEROS/AUXIN REPONSE FACTOR5 (MP/ARF5) [31]. Both *TMO5* and *T5L1* are expressed in the vasculature of the embryo and in the xylem precursor cells in the root meristem [31]. The *tmo5 t5l1* double mutants are impaired in periclinal vascular cell divisions and developed less vascular tissue in the roots [31]. Higher order mutants of genes in the *TMO5* clade showed more severe vascular tissue defects [31]. Our observations that gain-of-function *abs5-1D* mutants developed more complex leaf vascular tissues are in line with this report, suggesting that ABS5/T5L1 may promote the formation of veins. TMO5 clade proteins form heterodimers with LONESOME HIGHWAY (LHW) clade bHLH transcription factors [31]. When ectopically expressed, the TMO5/LHW dimer is able to induce periclinal cell divisions in non-vascular cells [31]. We showed that the curly leaf phenotype in *abs5-1D* is probably due to mis-coordinated growth of the adaxial and abaxial sides of the leaf. Since ABS5/T5L1 alone is not able to activate reporter gene expression, its activity may depend on the availability of its partners, such as the LHW proteins. One possibility for the “curled up” leaf phenotype of *abs5-1D* might be that the expression domains of LHW proteins in leaves are not evenly distributed on the abaxial and adaxial sides of leaves. Alternatively, there might be additional pathways that regulate differential growth of the adaxial and abaxial sides of leaves.

In this study, we show that *ABS7* encodes MYB101, which is a member of a small group of Arabidopsis *MYB* genes called the *GAMYB*s [38], [49], [50]. First identified in barley, *GAMYB* was named so for its involvements in phytohormone gibberellin (GA) mediated processes [49]. Studies in cereals and Arabidopsis have shown that *GAMYBs* are essential for GA-mediated programmed cell death in aleurone tissues during seed germination and in tapetum during anther maturation [50]–[53]. A recent report showed that *ABS7*/*MYB101*, as well as two other Arabidopsis *GAMYBs*, *MYB97* and *MYB120*, are highly expressed in mature pollen grains and pollen tubes and three genes share redundant functions in regulating proper pollen tube reception [54]. Several Arabidopsis *GAMYBs*, particularly *MYB33* and *MYB65*, are direct targets of *miR159* family microRNAs [50], [55]. However, *ABS7*/*MYB101* is not likely to be regulated by *miR159a*/*b*, because its expression pattern is not changed in any of the *miR159* mutant combinations and the sequence of putative microRNA targeting site in *ABS7*/*MYB101* is slightly different from those of *MYB33* and *MYB65* [50], [55]. Interestingly, loss of both *miR159a* and *miR159b* or the over-expression of a mutant form of *MYB33* with an abolished *miR159* targeting site results in a curled-up leaf phenotype that is similar to that of *abs7-1D*, suggesting that *MYB33* and *MYB65* might share similar functions with *ABS7*/*MYB101* and these functions are normally suppressed by *miR159s* [50]. In line with previous findings, we showed that ABS7/MYB101 likely functions as a transcription activator via yeast trans-activation assay [54]. Although normally *ABS7*/*MYB101* transcripts do not accumulate in leaves, our findings showed that mis-expressed *ABS7*/*MYB101* is able to regulate leaf morphology, possibly through the activation of down-stream target genes and the lack of regulation of *ABS7*/*MYB101* transcripts accumulation by *miR159* in leaves.

Lastly, we found that epidermal-specific expression of *ABS5*/*T5L1* or *ABS7*/*MYB101* driven by the *AtML1* promoter was sufficient to cause upwardly curly leaves and epidermal-specific *ABS5*/*T5L1* expressions can alter leaf auxin homeostasis (Figure 8). Our findings are consistent with previous studies that the leaf epidermis plays important roles in organ shape determination and plant development [42], [56], [57]. For example, epidermal-specific expression of brassinosteroid biosynthesis, signaling or inactivating genes are sufficient to promote or restrict the growth of the whole plant [56]. Leaf margin development provides another example of the involvement of epidermis in regulating plant organ morphogenesis [57]. Recent evidence suggests that mesophyll cells are also involved in the epidermal control of leaf development. Arabidopsis *ANGUSTIFOLIA3* (*AN3*) gene, encoding a transcription co-activator, has been identified as a critical mobile factor in coordinating leaf epidermal and mesophyll cell proliferation [58]. *AN3* transcripts can only be detected in the mesophyll layer, yet AN3 protein is able to move between the epidermal layer and the mesophyll layer [58]. Retaining AN3 protein in the mesophyll layer failed to complement the leaf development defects in *an3* mutant, indicating the inter cell layer movement of AN3 is essential to ensure proper leaf morphogenesis [58]. We did not determine the possibility of inter-cellular mobility for ABS5/T5L1 and ABS7/MYB101. However, our findings show leaf curvature can be manipulated through the epidermis alone and reinforce the notion that the epidermis plays important roles in leaf development.

## Materials and Methods

### Plant Materials and Growth Conditions

Wild type Arabidopsis and all mutants used in this study are in the Columbia-0 background. Arabidopsis seeds were sown on commercial soil mix (Pindstrup, Denmark) and stratified for two days at 4°C before placed in a growth room maintained at approximately 22°C under continuous illumination (∼100 µmol·m^−2^·s^−1^).

Transposon insertional line SM_3_20727 and T-DNA line SALK_146872C were obtained from the Arabidopsis Biological Resource Center (ABRC). The precise sites of transposon or T-DNA insertions were reconfirmed by sequencing PCR products that span both plant and foreign DNAs. All primers used in this study are listed in Table S1.

### DNA and RNA Techniques

Genomic DNA isolation, southern blot and northern blot analyses were carried out as described [59]. Total cellular RNAs were purified with Trizol Reagents (Life Technologies, USA). cDNAs used in semi-quantitative RT-PCRs were synthesized from 1 µg DNase I treated total RNA using PrimeScript II kit (TakaRa, Japan). Primers used in RT-PCRs are listed in Table S1.

### Histological Analysis

The middle region of the eighth rosette leaf of wild type and *abs5-1D* were hand cut and fixed in 4% (v/v) glutaraldehyde in 0.1 mM sodium phosphate buffer, pH 6.8, for 12 hours at 4°C. After fixation, samples were dehydrated in ethanol-xylene series and embedded in Paraplast (Sigma, St. Louis, MO, USA). Transverse leaf sections (10 µm) were prepared with Leica RM2265 microtome, mounted on glass slides, gradually deparaffinized and stained with safranin (0.5%, w/v) and fast green (0.5%, w/v) solutions.

To measure the number and length of leaf epidermal cells, leaf transverse sections were first photographed with DM5000B microscope (Leica) equipped with a CCD camera. Measurements were made using the LAS (Leica) software. Means and standard deviations were calculated from triplicate biological samples. Two-tailed Student's *t*-test was used to evaluate whether the measurements of *abs5-1D* or *abs7-1D* were significant different from those of wild type.

To test the impact of *abs5-1D* on *DR5::GUS* expression, homozygous *abs5-1D* plants were crossed with homozygous *DR5::GUS* plants and F1 plants were assayed for GUS activities. F1 plants of crosses between WT and *DR5::GUS* plants served as control. To test the effect of *ABS5* over-expression in *DR5::GUS* background, vector pBI111L-68810 was transformed into *DR5::GUS* homozygous lines and transgenic lines were obtained and assayed for GUS activities. Histochemical GUS assays were performed as described in [29].

### Vector Constructions and Transformations

Full-length cDNAs of *ABS5*/At1g68810 and *ABS7*/At2g32460 were amplified with primers 68810F & 68810R and 32460F & 32460R, respectively. The amplified fragments were ligated into pBluescript (pBS) and sequenced. To generate over-expression constructs, cDNA fragments of *ABS5*/*T5L1* and *ABS7*/*MYB101* were subcloned into a binary vector pBI111L and placed under the control of the constitutive 35 promoter to generate pBI111L-68810 and pBI111L-32460, respectively [60].

To generate a binary vector containing the epidermis-specific *AtML1* promoter, an *AtML1* promoter region of 3384 bp was amplified from wild type Arabidopsis genomic DNA as described in [43] and cloned into the *Hind*III and *Bam*HI sites of pBS for sequence confirmation. Next, the *AtML1* promoter was subcloned into a modified pBI111L vector that had the 35S promoter removed with *Hind*III and *Bam*HI digestions. The resulting construct was named pAtML1. The ORFs of *eGFP*, *ABS5* and *ABS7* were next cloned into the *Bam*HI site to generate *P_AtML1_::GFP*, *P_AtML1_::ABS5* and *P_AtML1_::ABS7*. Young leaves from *P_AtML1_::GFP* lines were embedded in 4% low melting agarose and 50 µm sections were examined via confocal microcopy (Olympus FV1000, Japan).

Transgenic plants were generated with the floral dip method and T1 transgenic plants were selected on half-strength Murashige and Skoog (MS) solid medium containing 50 mg·L^−1^ kanamycin [61].

### Arabidopsis Leaf Protoplast Transient Expression Assays

To generate a C-terminal GFP-tagged ABS5/T5L1, the coding sequences of ABS5/T5L1 was amplified with primers 68810F and 68810GFPR and subcloned into base vector pTF486 [59]. The resulting construct was designated *P_35S_*::*ABS5-GFP*. *P_35S_::ABS7-GFP* was similarly constructed with primers 32460F and 32460GFPR.

Leaf protoplast transformation and Hoechst33342 staining were performed as described in [28]. Bright field images and fluorescent signals from Hoechst33342, GFP and chlorophyll autofluorescence were monitored using a Leica DM5000B fluorescent microscope (Leica, Germany).

### Yeast Trans-activation Assays

For transcriptional activation activity assays, ORFs of *ABS5*/*T5L1* and *ABS7*/*MYB101* were cloned into the pGBKT7 vector (pBD), which contains the GAL4 DNA binding domain, to generate pBD-ABS5 and pBD-ABS7, respectively. The empty pGBKT7 vector was used as a negative control and the Arabidopsis *WRKY33* gene was included as a positive control [41]. Yeast strain AH109 was used. Each of the BD vectors was co-transformed with an empty AD vector, pGADT7. Yeast transformation and reporter gene activities were assayed according to manufacturer's instructions (Clontech, USA).

## Supporting Information

Figure S1 **Statistical analysis of the average number and length of epidermal cells of wild type,** ***abs5-1D*** **and** ***abs7-1D*** **.**
(TIF)

Figure S2 **Identification of a loss-of-function mutant allele of** ***ABS5*** **/** ***T5L1*** **.**
(TIF)

Figure S3 **Identification of a loss-of-function mutant allele of** ***ABS7*** **/** ***MYB101*** **.**
(TIF)

Figure S4 **Accumulation of** ***ICU1-4*** **transcripts in wild type, loss-of-function and activation-tagged lines of** ***ABS5*** **/** ***T5L1*** **and** ***ABS7*** **/** ***MYB101.***
(TIF)

Table S1 Primers used in this study.
(PDF)
