# Supplementary figures and images for: The Over-Expression of Two Transcription Factors, ABS5/bHLH30 and ABS7/MYB101, Leads to Upwardly Curly Leaves
Source: PLoS One. 2014 Sep 30;9(9):e107637. doi: 10.1371/journal.pone.0107637 (PMC4182325; doi:10.1371/journal.pone.0107637)

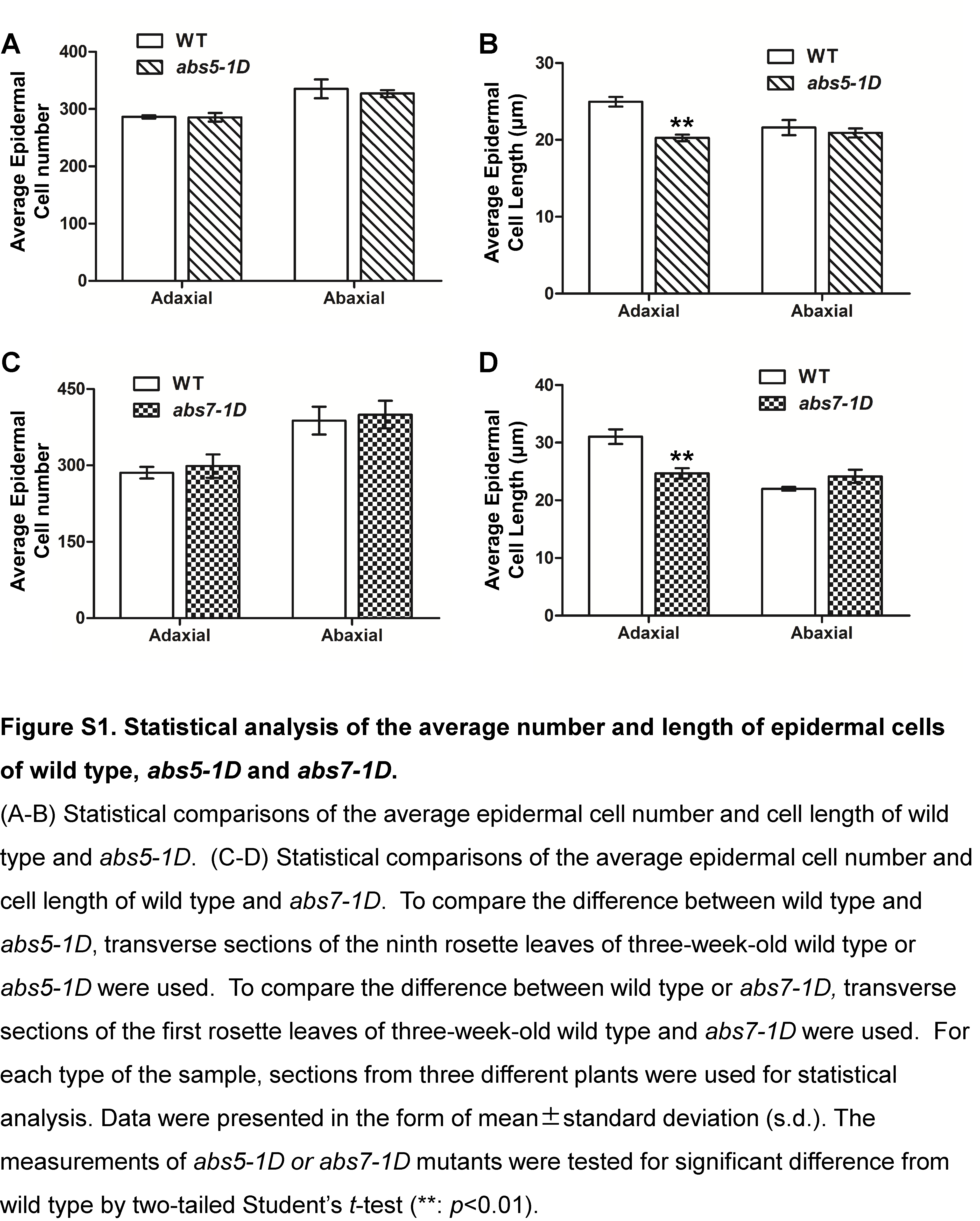

Supplement: Figure S1 — Statistical analysis of the average number and length of epidermal cells of wild type, abs5-1D and abs7-1D . (TIF) [file pone.0107637.s001.tif]

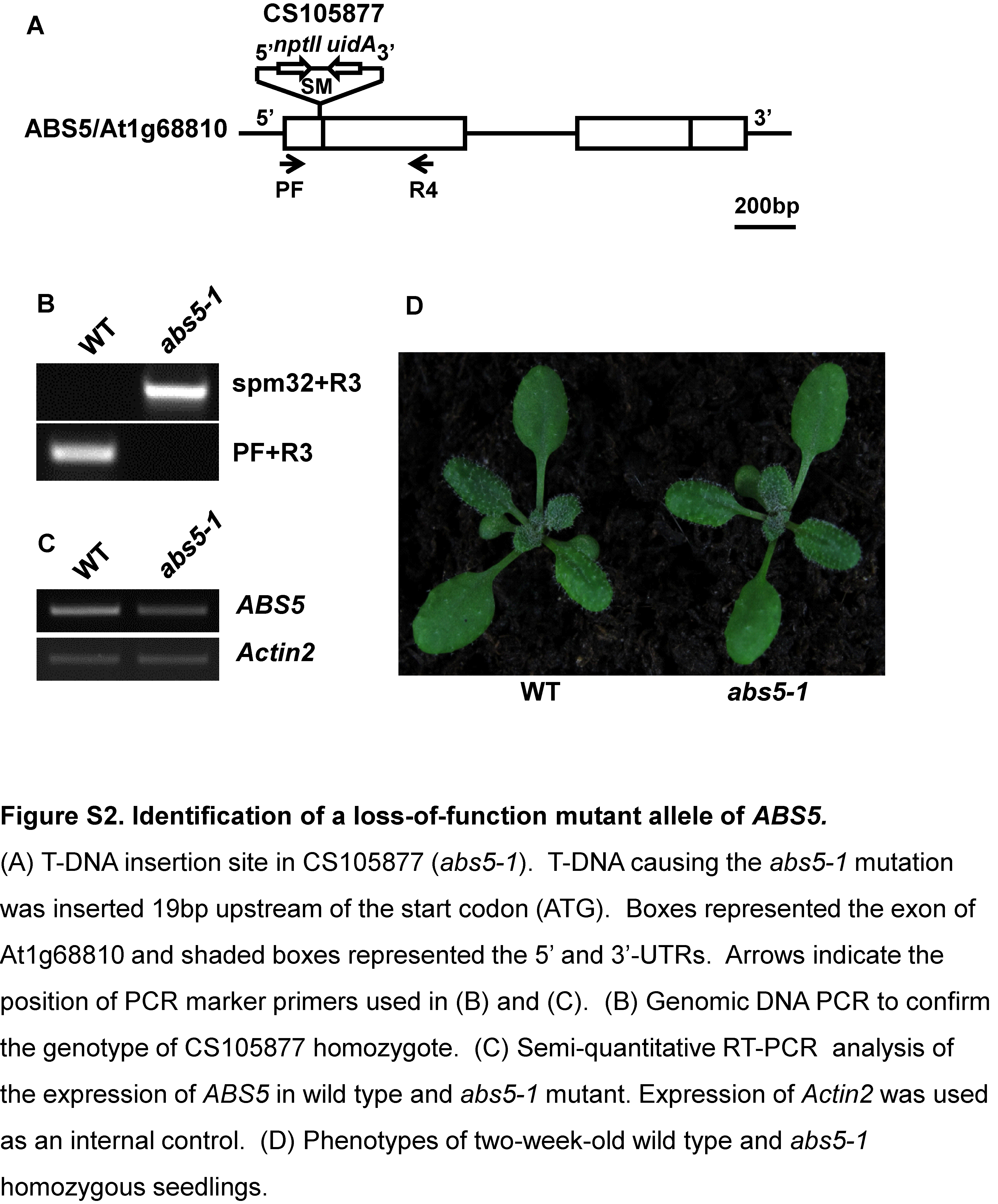

Supplement: Figure S2 — Identification of a loss-of-function mutant allele of ABS5 / T5L1 . (TIF) [file pone.0107637.s002.tif]

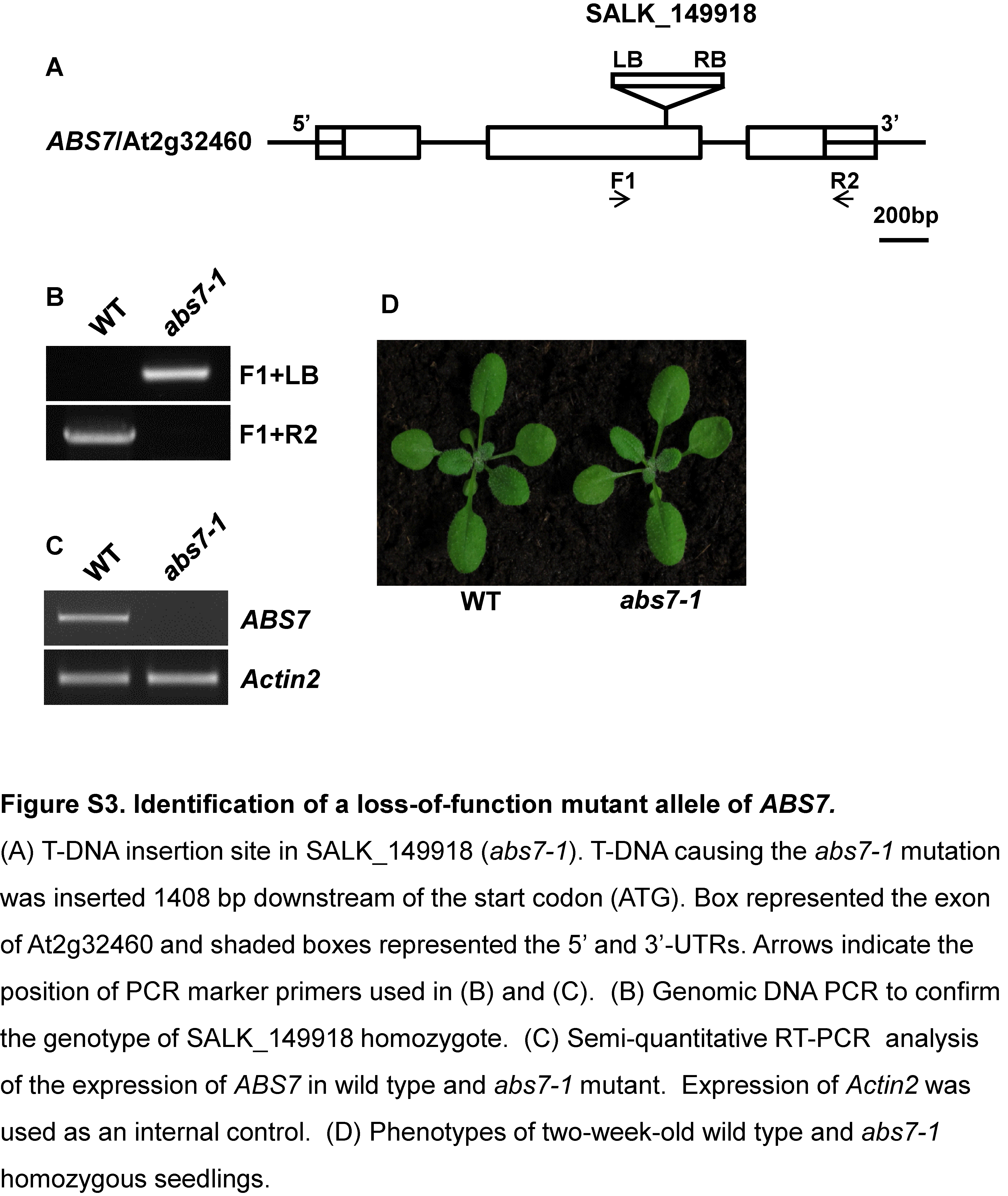

Supplement: Figure S3 — Identification of a loss-of-function mutant allele of ABS7 / MYB101 . (TIF) [file pone.0107637.s003.tif]

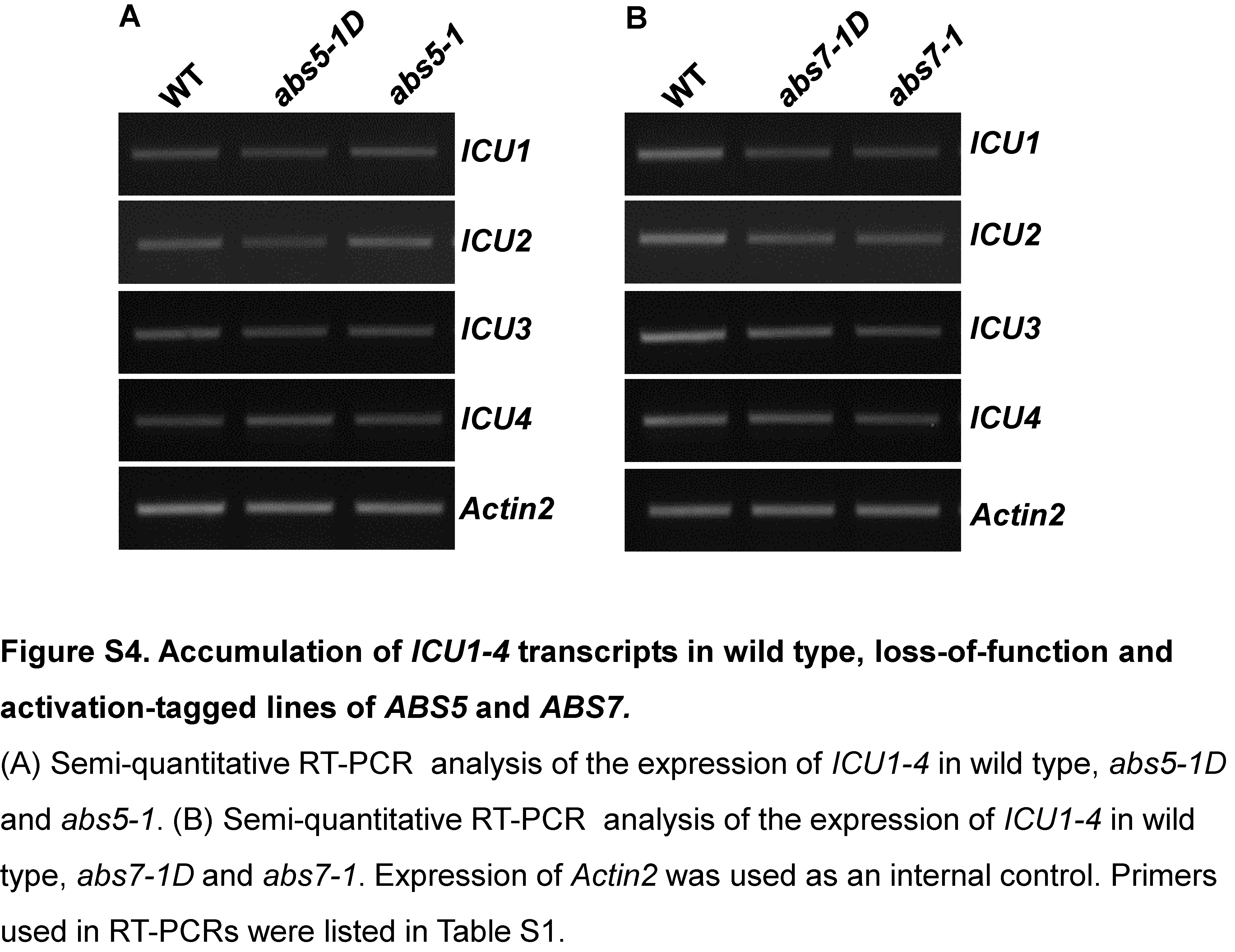

Supplement: Figure S4 — Accumulation of ICU1-4 transcripts in wild type, loss-of-function and activation-tagged lines of ABS5 / T5L1 and ABS7 / MYB101. (TIF) [file pone.0107637.s004.tif]
