# Supplementary material for: The Over-Expression of Two Transcription Factors, ABS5/bHLH30 and ABS7/MYB101, Leads to Upwardly Curly Leaves
Source: PLoS One. 2014 Sep 30;9(9):e107637. doi: 10.1371/journal.pone.0107637 (PMC4182325; doi:10.1371/journal.pone.0107637)
Supplement: Table S1 — Primers used in this study. (PDF) [file pone.0107637.s005.pdf]

Table S1. Primers used in this study.

Primer Name	Primer sequences	Notes
68810F	5'-CATGGATCCTAGAGAGAGAGAAGAGGACAAAG-3'	cDNA cloning
68810R	5'-CATGGATCCCGTGACGTACAACATTCATTTCC-3'	
68810GFPR	5'-CATGCCATGGAACCACCACCACCACCACCCCTCT GATTATATTGTTGTTG-3'	P_{35S}::ABS5-GFP vector construction
68810F1	5'-AATCATCTCGCTAAGCTCCG-3'	RT-PCR
68810R1	5'-CCTCAATCGTCCCTATACAG-3'	
68810F2	5'-CATCATATGTGCGCTAAGAAAGAAG-3'	pBD-ABS5 vector construction
68810R2	5'-CATGAATTCTTACCTCTGATTATAT-3'	
68810PF	5'-CGCATTGCTTGTCCATATAC-3'	*abs5-1* genotyping
68810R3	5'-CATGGTACCGAAACCAGATCCAACGTTAC-3'	
spm32	5'-TACGAATAAGAGCGTCCATTTTAGAGTGA-3'	
32460F	5'-CATGGATCCGTTGAAAAGGATGGATGGTGGTG-3'	cDNA cloning
32460R	5'-CATGGATCCACATACAGTTCATAGCACATCCC-3'	
32460GFPR	5'-CATGGGATCCACCACCACCACCACCACAGATGCT AGGCATGTTGCT-3'	P_{35S}::ABS7-GFP vector construction
32460F1	5'-ACTAGACAATAGCGTCCTAG-3'	RT-PCR
32460R1	5'-TGTTGCTCCAATAGCATGAC-3'	
32460F2	5'-CATCCATGGGGATGGATGGTGGTGGAGAGA-3'	pBD-ABS7 vector construction
32460R2	5'-CATGGATCCCTAACAGATGCTAGGCATGTT-3'	
Salk_LB	5'-GAACAACACTCAACCCTATCTC-3'	*abs7-1* genotyping
23380F1	5'-GAGGTCTTCTATGGAACGAG-3'	*ICU1* gene RT-PCR
23380R1	5'-AGTACCTGCACCATCTGATG-3'	
67100F1	5'-ACTATCACTGGTGAAGGTAG-3'	*ICU2* gene RT-PCR
67100R1	5'-GAGCTGTAACAATTCCTGAG-3'	
05040F1	5'-CAGTGATGCTTCTACTGCAG-3'	*ICU3* gene RT-PCR
05040R1	5'-CTGTCCATCACTGCTACATG-3'	
52150F1	5'-TTCTCATGGTTGCACTGGTG-3'	*ICU4* gene RT-PCR
52150R1	5'-TCTCCTATCACTGACCATCC-3'	
